# Distance to Multidisciplinary Team Clinic in Gaborone, Botswana, and Stage at Cervical Cancer Presentation for Women Living With and Without HIV

**DOI:** 10.1200/GO.22.00183

**Published:** 2022-11-17

**Authors:** Tara M. Friebel-Klingner, Lisa Bazzett-Matabele, Doreen Ramogola-Masire, Barati Monare, Tlotlo B. Ralefala, Alexander Seiphetlheng, Gaobakwe Ramontshonyana, Peter Vuylsteke, Nandita Mitra, Douglas J. Wiebe, Timothy R. Rebbeck, Anne Marie McCarthy, Surbhi Grover

**Affiliations:** ^1^ Department of Biostatistics, Epidemiology and Informatics, Perelman School of Medicine, University of Pennsylvania, Philadelphia, PA; ^2^ Botswana-University of Pennsylvania Partnership, Gaborone, Botswana; ^3^ Department of Obstetrics and Gynecology, Faculty of Medicine, University of Botswana, Gaborone, Botswana; ^4^ Department of Obstetrics and Gynecology, Yale University School of Medicine, New Haven, CT; ^5^ Department of Oncology, Princess Marina Hospital, Gaborone, Botswana; ^6^ University of Botswana, Gaborone, Botswana; ^7^ Department of Internal Medicine, Faculty of Medicine, University of Botswana, Gaborone, Botswana; ^8^ Dana-Farber Cancer Institute and Harvard TH Chan School of Public Health, Boston, MA; ^9^ Department of Radiation Oncology, University of Pennsylvania, Philadelphia, PA

## Abstract

Cervical cancer is the leading cause of cancer death for women in Botswana. Barriers in access to cancer care can lead to later stages at diagnosis and increased mortality. This study evaluated access, defined as travel time from a patient's residential village to a multidisciplinary team clinic in Gaborone, with stage of cervical cancer at presentation. In addition, because of the high HIV prevalence in Botswana, we explored the association between travel time and HIV status.

**METHODS:**

Eligible patients with cervical cancer presenting to the multidisciplinary team between 2015 and 2020 were included. Data were abstracted from questionnaires and hospital records. Google Maps was used to calculate travel time. Multinomial regression was used to examine travel time and cancer stage, and multivariable logistic regression was used to investigate travel time and HIV status.

**RESULTS:**

We identified 959 patients with cervical cancer of which 70.1% were women living with HIV. The median travel time was approximately 2 hours. Using a reference group of stage I disease and a travel time of < 1 hour, the odds of presenting with stage II increased for patients traveling 3-5 hours (adjusted odds ratio [OR], 2.00; 95% CI, 1.14 to 3.52) and > 5 hours (OR, 2.19; 95% CI, 1.15 to 4.19). There were no significant associations for stage III. For stage IV disease, the odds were increased for patients traveling 3-5 hours (OR, 2.93; 95% CI, 1.26 to 6.79) and > 5 hours (adjusted OR, 4.05; 95% CI, 1.62 to 10.10). In addition, the odds of patients presenting living with HIV increased with increasing travel time (trend test = 0.004).

**CONCLUSION:**

This study identified two potential factors, travel time and HIV status, that influence access to comprehensive cervical cancer care in Botswana.

## INTRODUCTION

In Botswana, cervical cancer is the most common cause of cancer and cancer death among women.^1,2^ Studies have shown that approximately 70% of women diagnosed with cervical cancer in Botswana are living with HIV.^3-5^ To curb this disease burden, particularly in women living with HIV (WLWH), the Botswana Ministry of Health^6,7^ adapted the American Society of Clinical Oncology's recommendations and implemented the Papanicolaou cytology test and visual inspection with acetic acid screening strategies.^8-10^

**CONTEXT**

**Key Objective**
How is distance to comprehensive cancer care associated with cervical cancer stage at presentation in a multidisciplinary team clinic in Gaborone, Botswana?
**Knowledge Generated**
This study found that approximately 50% of patients with cervical cancer in Botswana traveled over 2 hours and more than 130 km to attend the multidisciplinary clinic. Patients with greater travel time had higher odds of presenting with later-stage disease and were more likely to be living with HIV.
**Relevance**
Our findings indicate that increased distance (ie, travel time) is a barrier to accessing comprehensive cancer care. Many cervical cancer cases in Botswana may miss being detected and are not presenting for comprehensive life-saving care. These findings can help guide future interventions aiming to improve cervical cancer outcomes for women in Botswana.

The standard-of-care treatment for the majority of presenting cervical cancers is radiotherapy. Botswana provides government-funded health care for its 2.3 million citizens^11^ but has only one radiation oncology facility with one linear accelerator and brachytherapy unit located in the capital city of Gaborone. In 2015, a multidisciplinary gynecological oncology team (MDT) clinic was formed at Princess Marina Hospital (PMH) to provide comprehensive cancer care for patients with cervical cancer presenting to the two referral hospitals in Gaborone: PMH and Gaborone Private Hospital.^12^

With limited resources, it is a challenge for women in Botswana to have equitable access to comprehensive cancer care. Studies have shown that barriers to access can cause a delay in presentation and can result in progression of a malignant tumor to a later stage with higher mortality rates.^5,13-16^ One aspect of access, travel time, has been associated with delays and late-stage cancer outcomes.^17,18^ Accessibility to care, defined by travel time, and its association with cancer stage have not yet been studied in Botswana.

For this study, we assessed the travel burden for patients with cervical cancer presenting to the MDT clinic for staging and treatment. We hypothesized that patients with a greater travel time from their village to the MDT clinic would be more likely to present with later-stage disease than patients with a shorter travel time. We also hypothesized that WLWH may be more likely to present for care despite a higher travel burden than women without HIV, given that cervical cancer prevention measures were initially implemented and prioritized for WLWH, who are at a four to six times increased risk of developing cervical cancer.^19^

## METHODS

This study included patients presenting to the MDT clinic in Gaborone during January 2015-March 2020, as described previously.^12,20^ Eligible patients were age ≥ 18 years and not pregnant, had a diagnosis of histopathologically confirmed cervical cancer, and provided written informed consent.

Each patient's residential village was abstracted from a questionnaire administered at the initial MDT consult. Using a geographic shapefile for Botswana,^21^ patients were linked to their respective village and geocoded using ArcMap 10.7.1 software (Esri, Redlands, CA) to the geographic centroid of their village.^22^ Latitude and longitude coordinates were obtained from the ArcMap geography feature.^22^ PMH coordinates were used for the MDT clinic.

Geographic proximity of village to the MDT was measured in travel time using Google Maps.^23,24^ Starting with the geographic coordinates of the village centroid, we imputed travel time in minutes (assuming car transport using road networks and travel routes) to the MDT clinic for each patient.^25^ We characterized travel time into four categories: < 1 hour (11-59 minutes), 1-3 hours (60-179 minutes), 3-5 hours (180-299 minutes), and ≥ 5 hours (≥ 300 minutes). Travel time was mapped and displayed on a choropleth map using ArcMap.^22^

We calculated presentation rates of cervical cancer cases for each village. We defined presentation rate as the number of patients with cervical cancer presenting to the MDT clinic from each village (numerator) divided by the population projection for females age ≥ 20 years for each village (denominator).^21^

The primary outcome of interest was stage of cervical cancer at presentation on the basis of the International Federation of Gynecology and Obstetrics staging system^13,26^ and categorized as stage I (IA, IA1, IA2, IB, IB1, IB2, and IB3), stage II (IIA and IIB), stage III (IIIA, IIIB, and IIIC), or stage IV (IVA and IVB). Our secondary outcome, HIV status, was based on clinical abstraction of medical records at the initial MDT visit.

Data were abstracted from questionnaires administered during the initial consult visit and from medical records. Data collected included sociodemographic and clinical factors (ie, place of residence/village, age, marital status, history of cervical cancer screening, ever/never visit with a traditional doctor and/or a natural healer, presence of abnormal vaginal bleeding, including postcoital bleeding/ bleeding after vaginal intercourse), and additionally, among WLWH, data associated with living with HIV (ie, status of antiretroviral treatment [ART] and CD4 count [> 250 cells/mL]). Urban or rural designation was based on the patient's geo-coded district of residence.^11^ We also obtained female HIV prevalence for each district using estimates from the Botswana AIDS Impact Survey IV.^27^

We examined variables of interest by travel time using Pearson's chi-square test, Student *t* tests, and ANOVA as appropriate. Variables were determined a priori on the basis of availability, purposeful selection, review of the literature, and clinical relevance. The explanatory variable of interest was travel time from village residence to MDT clinic.

We used multinomial (polytomous) logistic regression to estimate odds ratios (ORs) for cervical cancer stage at presentation (stages II, III, and IV) compared with the reference stage (stage I) by travel time. We ran univariate and multivariable regression models adjusted for potential confounders and performed tests for trend.^20^ We also investigated the interaction of travel time with HIV status with respect to stage at presentation and repeated the analysis stratified by HIV status. To assess the association of HIV status and travel time, we used logistic regression models, both unadjusted and adjusted for a priori selected variables. Analyses were conducted using STATA 16 (StataCorp, College Station, TX). A *P* value < .05 was considered statistically significant.

### Ethics Approval

This study was approved by the University of Pennsylvania as part of the Botswana-University of Pennsylvania Partnership (IRB: 820159 IRB#7 Penn) and by the Ministry of Health of the Republic of Botswana (HPDME 13/18/1).

## RESULTS

**FIG 1:**
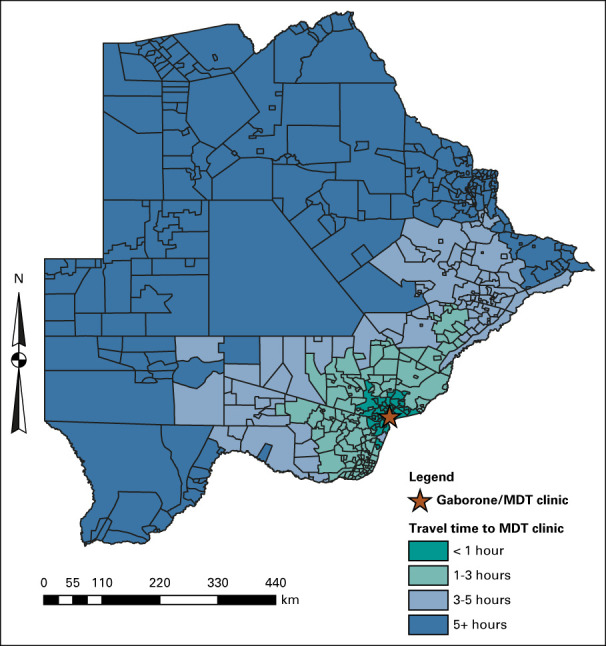
Distribution of travel time from villages throughout Botswana to the MDT clinic in Gaborone. MDT, multidisciplinary team.

During January 2015-March 2020, 1,019 patients presented to the MDT clinic with cervical cancer. We excluded 19 patients (1.8%) diagnosed with cervical carcinoma in situ, seven patients (0.7%) with recurrent disease, and 34 patients (3.3%) missing village of residence. Sociodemographic and clinical characteristics of the 959 eligible patients are presented in Table 1. Most patients presented with stage III disease (37.7%, n = 332) and were WLWH (70.1%, n = 660). The mean age was 50.7 (range: 22.4-95.2) years. The median travel time was 124.0 (interquartile range: 54.1-280.0) minutes, and the median distance was 134.7 (interquartile range: 52.8-392.9) km. Figure 1 depicts travel time categories for villages to the MDT clinic.

**TABLE 1:**
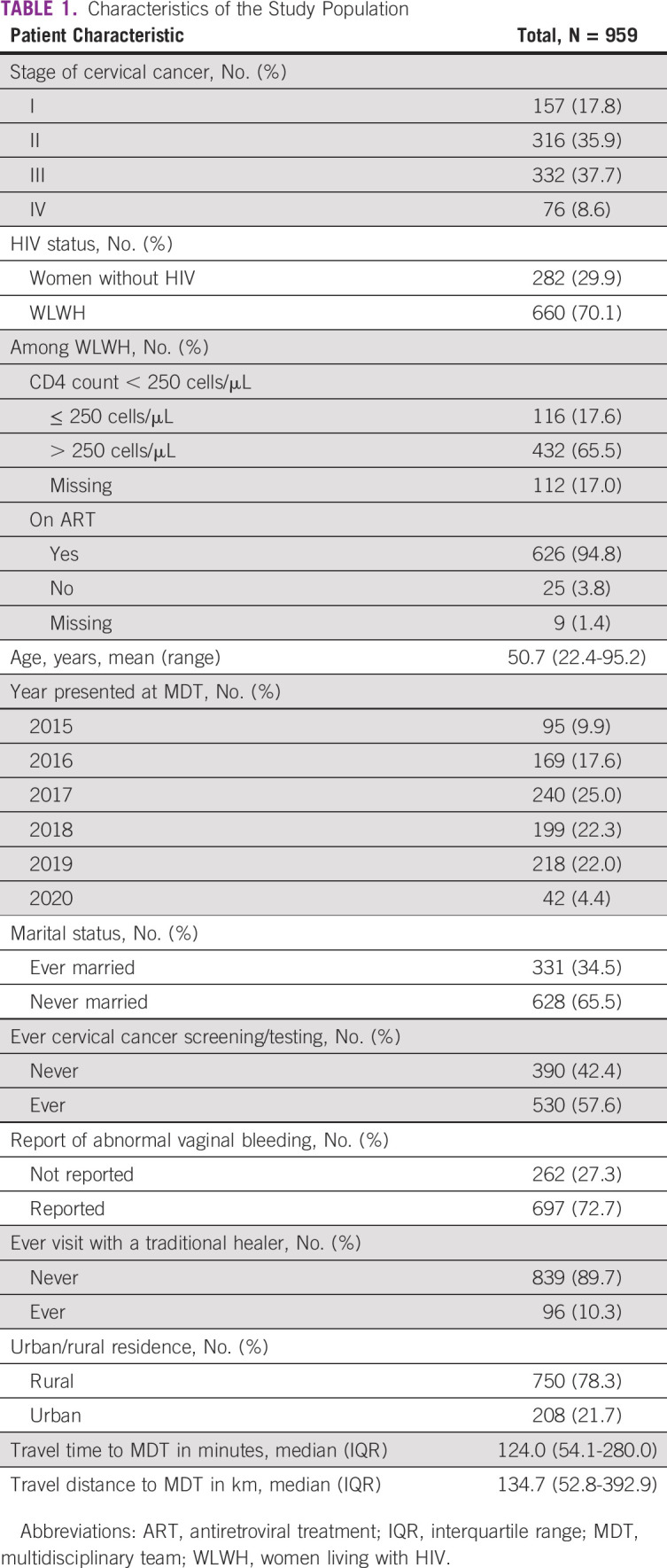
Characteristics of the Study Population

Table 2 shows patient characteristics by travel time categories. Across categories, patients differed in stage at presentation (*P* = .016), with more patients with stage I cancers from areas within 1 hour of travel time (21.3%) versus 5 hours (11.8%), and conversely, patients with stage IV cancers more often presented from villages over 5 hours away (11.2%) versus < 1 hour (5.3%). More WLWH presented from areas with more than 5 hours of travel time versus areas within 1 hour (78.6% *v* 72.1%, respectively; *P* < .001). Women tended to be older (mean: 52.9 years) from areas with 1-3 hours of travel time than women from 3 to 5 hours of travel time (mean age: 49.8; *P* = .020). In addition, most women from urban areas were within 1 hour of travel time versus 5 hours of travel time (38.3% *v* 0.6%, *P* < .001). Among WLWH, more women with higher travel times were on ART (98.3% *v* 93.8%, *P* = .022), but there was no difference by CD4 count. There were also no differences across travel time for marital status, cervical cancer screening, abnormal vaginal bleeding, or visits with a traditional healer.

**TABLE 2:**
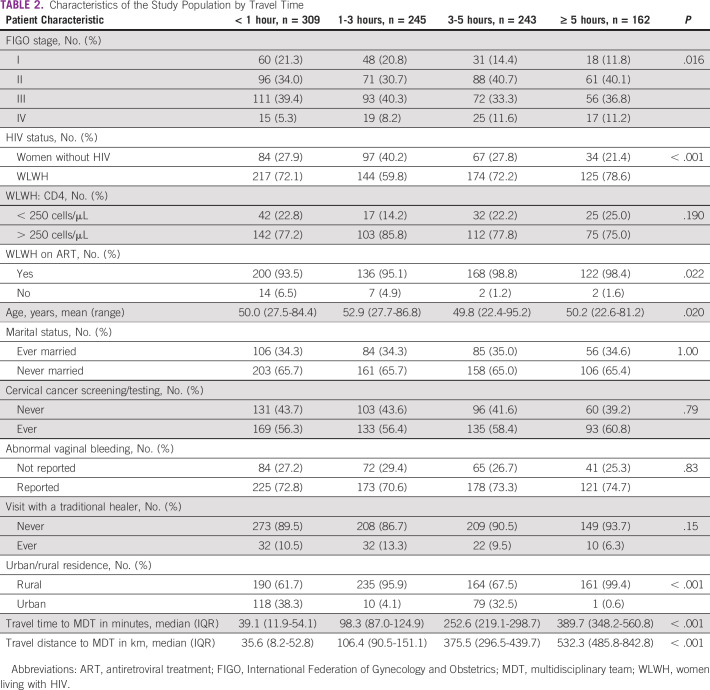
Characteristics of the Study Population by Travel Time

**FIG 2:**
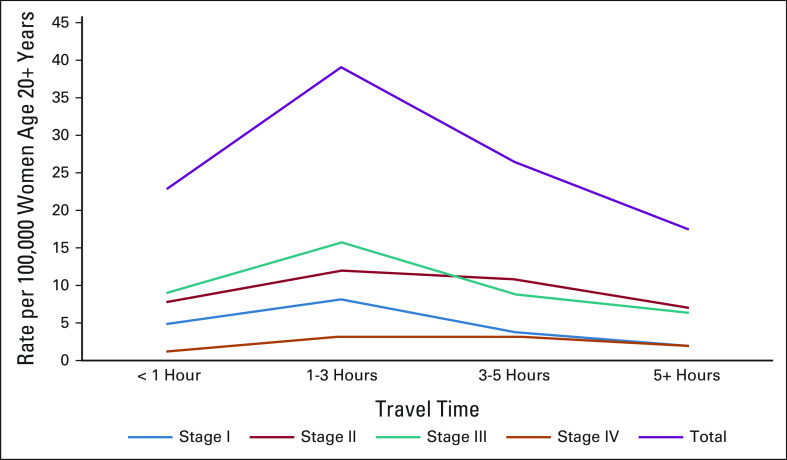
Presentation rates per 100,000 women age ≥ 20 years by cancer stage from 2015 to 2020.

Figure 2 depicts presentation rates for cervical cancer cases as nonlinear but overall indicates a decline with increasing time to clinic. Rates were highest for patients traveling 1-3 hours (39.1 per 100,000 women) and lowest for patients traveling over 5 hours (17.5 per 100,000).

The odds of presenting with later-stage cancer increased with increasing travel time (Table 3). Using a reference group of stage I cancer and a travel time of < 1 hour, the odds of presenting with stage II doubled (adjusted OR [aOR], 2.00; 95% CI, 1.14 to 3.52) for patients traveling 3-5 hours and more than doubled for patients traveling > 5 hours (aOR, 2.18; 95% CI, 1.10 to 4.31; trend test *P* < .001). There was no significant trend, nor any difference by travel time, observed among those presenting with stage III disease versus stage I. The odds of presenting with stage IV versus the reference group nearly tripled when traveling 3-5 hours (aOR, 2.97; 95% CI, 1.28 to 6.90) and more than quadrupled for patients traveling > 5 hours (aOR, 4.26; 95% CI, 1.61 to 11.30; trend test *P* < .001). We did not observe a significant interaction between travel time and HIV status with respect to stage at diagnosis (*P* = .155). For the HIV-stratified analysis (Appendix Tables A1 and A2), we observed similar results to the main analysis for WLWH, but there were no significant results for women without HIV, except for the trend test for women living over 5 hours away with stage IV cancer. Null results in this group could be due to the lack of power in the smaller sample (n = 268).

**TABLE 3:**
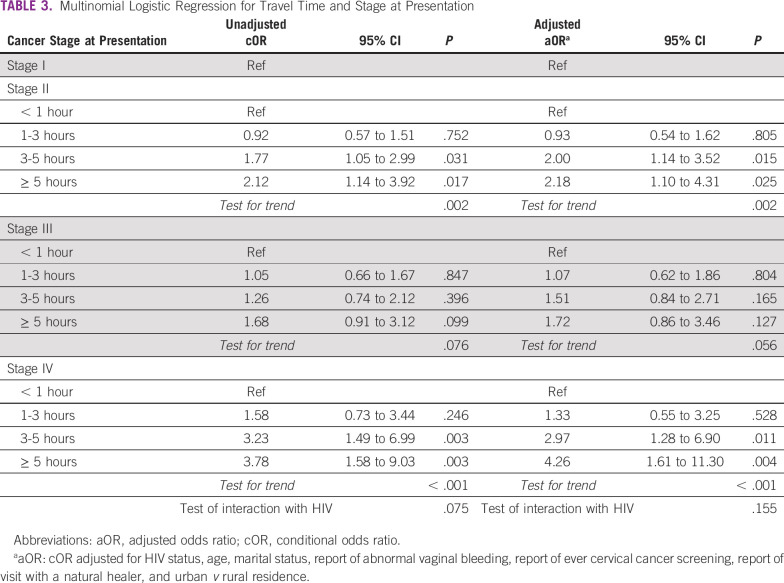
Multinomial Logistic Regression for Travel Time and Stage at Presentation

The proportion of presenting cervical cancer cases by HIV status differed by travel time to the MDT clinic (Table 4). The female HIV prevalence was analogous across all time categories (range: 20.4-21.1); however, the proportion of WLWH presenting with cervical cancer was lowest for patients traveling 1-3 hours (59.8%) compared with women traveling > 5 hours (78.6%). The odds increased for WLWH with increasing travel times (*P*-trend = .002). Patients living ≥ 5 hours away were twice as likely to be living with HIV as patients traveling < 1 hour (aOR, 2.03; 95% CI, 1.10 to 3.75).

**TABLE 4:**
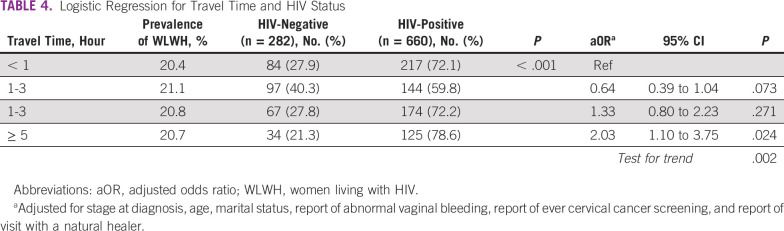
Logistic Regression for Travel Time and HIV Status

## DISCUSSION

This study delineates travel time and its association with cervical cancer stage for patients traveling from their village of residence to the comprehensive MDT cancer clinic in Gaborone, Botswana. Approximately 50% of patients traveled over 2 hours and more than 130 km to attend the MDT clinic. Patients with greater travel time had higher odds of presenting with later-stage disease. The presentation rate of cervical cancer cases to the MDT clinic also differed by travel time, with the highest rate (39.1 per 100,000 women) in patients traveling 1-3 hours over the 4.5-year study period. Rates from GLOBOCAN for South Africa estimate 36.4 new cervical cancer cases per 100,000 women per year,^28^ and the Botswana National Cancer Registry^1^ estimates approximately 374 new cases per year. Our study averaged approximately 215 cervical cases per year over 4.5 years, and thus, our estimates fall well below the expected number of patients with cervical cancer, particularly in areas more than 3 and 5 hours away from Gaborone. This finding could indicate that many cervical cancer cases in these areas may miss being detected and thus are not presenting for comprehensive life-saving care.

Our results also found that in areas with increased travel time, women were more likely to be living with HIV and on ART, suggesting that living with HIV and having it well-managed may increase the likelihood of seeking cervical cancer care despite a higher travel burden. Understanding that cervical cancer prevention measures were initially implemented and prioritized specifically for WLWH in Botswana who are at higher risk of developing cervical cancer,^19^ WLWH are likely to have increased access to health care and might have increased health-seeking behaviors. We also observed a higher proportion of women on ART than is reported in the general population.^3^ This further supports the conclusion that WLWH on treatment have increased access to health care and may be more likely to present than women without HIV or WLWH who are not on treatment and much sicker.

Increased travel burden is a risk factor for poor health outcomes, including later stage at cancer presentation.^17,18,29-32^ In a systematic review of more than 108 studies, 77% of studies showed that greater distances to care are associated with poorer health outcomes.^17^ In accordance with the literature, our study also shows that increased travel time is associated with increased odds of presenting with later stages of cervical cancer. Travel time is only one aspect of access to care, and later stage at presentation is likely a result of multiple patient-level and health system delays that may be exacerbated at greater distances. Access is multidimensional and consists of availability, accessibility, accommodation, affordability, and acceptability.^32^ Living farther away from comprehensive cancer care in a resource-limited setting understandably increases barriers to all aspects of access.

A recent review of 15 studies in low- and middle-income countries^34^ reports that lack of knowledge of cervical cancer is the most prevalent barrier to cervical cancer screening. Two studies in Botswana^35,36^ report that lack of understanding of both risk factors for cervical cancer and the importance and benefits of screening is a barrier to accessing cervical cancer screening. Indeed, failure to identify the need to screen and counsel is the first potential failure during cancer care.^37^ This could be particularly true for women living further from treatment facilities in more rural and remote areas, where women and health care providers may be unaware of the need to screen for cervical cancer and may lack knowledge regarding related symptoms. Other studies in sub-Saharan Africa have also noted more advanced disease in rural patients compared with urban patients.^38-40^ These burdens, together with the cost and feasibility of travel, all contribute to delays in access and fewer patients presenting for care.

The presentation rate of cervical cancer cases to the MDT clinic also differed by travel time. Areas with lower presentation rates could indicate a lack of detection of cervical cancer cases, and thus, not all patients with cervical cancer are presenting for appropriate care and treatment. Furthermore, rates of women presenting for care were not linear in relation to travel time, likely reflecting the influence of several factors on access.

As previously mentioned, rural areas over 5 hours away have a high travel burden and likely encounter multiple barriers to access. However, areas within 1 hour of travel time had lower presentation rates than areas 1-3 hours away. Although travel time may be less of a barrier for areas within 1 hour of Gaborone, multidimensional access issues could also be at play.^33^ In the urban area surrounding the MDT, there could be a disproportionate supply and demand for health services that could contribute to lower presentation rates.^41^ Further investigation into other barriers of access in this area would be informative.

The proportion of WLWH presenting to care increased with increasing distance from the MDT clinic. Given that the prevalence of WLWH was similar by travel time/geographic area,^27^ we would not expect risk factors for human papilloma virus/HIV infection to differ drastically across locations, and therefore, we would not expect differences in rates to be driven by underlying differences in biology or incidence. Instead, our results may provide insight into the influence of HIV status when accessing health care. In the wake of the HIV epidemic, several steps were taken to prioritize cervical cancer prevention, particularly in WLWH, who are four to six times more likely to develop cervical cancer than women without HIV,^42^ and thus, WLWH might have greater knowledge of and exposure to cervical cancer screening.^43^ There could also be health system–level effects, where providers recognize cervical cancer screening as particularly beneficial for high-risk WLWH. In addition, more than 80% of people living with HIV in Botswana are on ART^27^ and thus have regular contact with health care providers and facilities. This increased interaction could provide WLWH with more resources and opportunities to test or screen for cervical cancer, as was also suggested in a previous study in Botswana investigating delays in cancer care.^44^ In addition, we did observe that significantly less women living closer to Gaborone than further away were on ART and that the proportion of women on ART in our sample was also higher than the average proportion across Botswana,^3^ which could indicate that WLWH who are sicker and not well managed may also not be presenting to the MDT clinic.

This study does have some limitations and challenges. No conclusions can be made about causality of the study variables on disease stage because of the retrospective study design. Given methodological challenges when using distance to care as an exposure for health outcomes, selection bias could influence our findings.^45^ Although our study is vulnerable to selection bias, we cannot accurately quantify the degree of bias because of under-reporting in the current Botswana population-based cancer registry.^1^ Although selection bias is a potential issue, identifying it is an important finding, as it may help identify women who are not presenting for appropriate cervical cancer treatment in Botswana. In our stratified analysis, for WLWH, results were similar in direction, magnitude, and significance to the main analysis, but for women without HIV, the results varied. Future studies with more power could investigate this association for women without HIV.

In addition, this study collected data at the time of presentation and is therefore subject to recall bias, social desirability bias, and potential unmeasured confounding.^45^ We were also unable to account for potential confounders including many socioeconomic factors that might be differential by location including socioeconomic status, education level, childcare needs, employment status, and health literacy although we were able to account for urban and rural status, which could be a proxy for some of these factors. There is also potential for misclassification of travel time because of use of the village centroid, but choosing the village centroid is a more systematic way to determine a location than using random points in the village.

Finally, this study examined only one metric of access, travel time, so it will be important for future studies to investigate additional aspects of access. Untangling differences in awareness, knowledge, screening, and health system factors along the cervical cancer care continuum will offer insight about where future interventions would be most effective and beneficial.

In conclusion, this study identified that increased travel time was associated with later-stage cervical cancer at presentation. In addition, our results suggest that WLWH are more likely to present for cervical cancer care. Identifying travel time as a barrier and HIV as a potential facilitator to cancer care can help guide future interventions aiming to improve cervical cancer outcomes for all women in Botswana.

## Figures and Tables

**TABLE A1:**
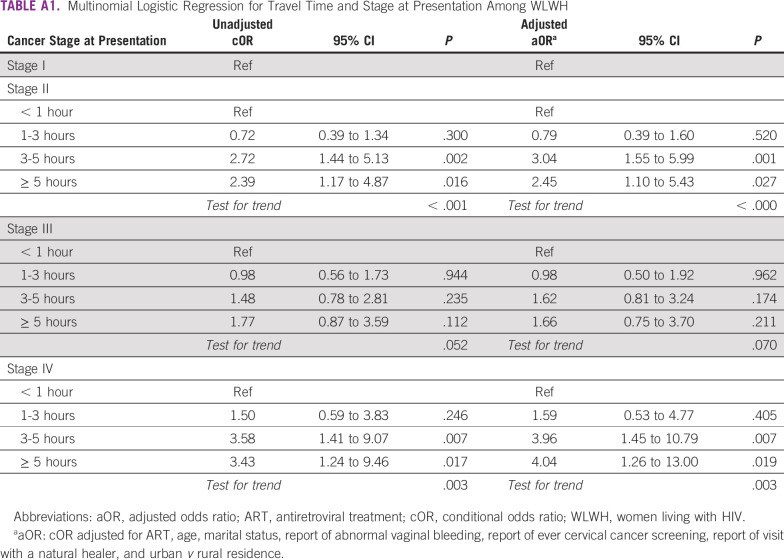
Multinomial Logistic Regression for Travel Time and Stage at Presentation Among WLWH

**TABLE A2:**
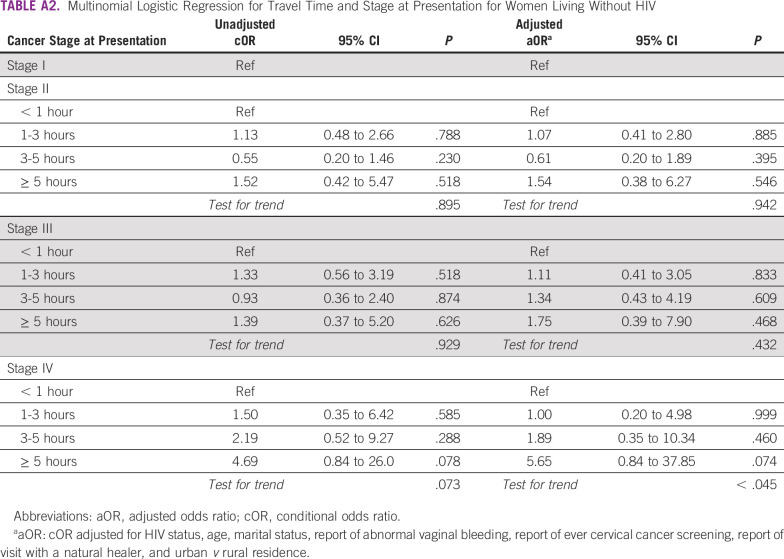
Multinomial Logistic Regression for Travel Time and Stage at Presentation for Women Living Without HIV

## Data Availability

The data sets generated and/or analyzed during the current study are not publicly available due to identifying information but are available from the corresponding author on reasonable request.
